# Acetanilide as a Nervine

**Published:** 1888-02

**Authors:** 


ACETANILIDE AS A NERVINE.—Influenced by the observations of Dujardin-Beaumetz, Dr. Seifert, of Würzburg, has essayed the use of acetanilide in various painful maladies. The results of this trial are given in a paper published in the *Wiener. med. Wochenschrift*, No. 35,

1887, an abstract of which is given in the *Centralblatt für die gesammte Therapie* for October, 1887. In two cases of migraine, it proved to be very effective in eight-grain doses, and it was also equally useful in six cases of general headache with anæmic basis. In three cases of trigeminal neuralgia, Dr. Seifert had excellent results. For the most part, his observations are in accord with those of Dujardin-Beaumetz.

